# Shipping Conditions May Impact the Reliability of At-Home Microsampling Devices

**DOI:** 10.1021/acs.jproteome.6c00117

**Published:** 2026-07-14

**Authors:** Andrea Villanueva Raisman, Jakob Naess, David Kotol, Fredrik Edfors

**Affiliations:** † SciLifeLab, Department of Protein Science, Division of Systems Biology, School of Chemistry, Biotechnology and Health, KTH Royal Institute of Technology, 171 65 Solna, Sweden; ‡ ProteomEdge AB, 106 91 Stockholm, Sweden

**Keywords:** microsampling, mass spectrometry, DBS

## Abstract

Targeted proteomics offers high precision, reproducibility, and multiplexing capabilities for quantifying proteins in complex biological samples, making the technology into a powerful tool for biomarker discovery and clinical diagnostics. When combined with at-home microsampling, this approach has the potential to transform population-level screening and longitudinal studies and can pave the way for decentralized healthcare. One of the main advantages of this combined approach is that samples can be collected offsite and then transported and stored as dried blood spots (DBSs). Currently, innovative volumetric microfluidic devices have also enabled DBS microsampling of consistently precise volumes. However, the reliability of the molecular data obtained with this mode of sampling still needs to be investigated and validated. In this study, 72 deidentified blood samples from two individuals were collected using DBS microsampling device CapitainerB and analyzed using targeted mass spectrometry and quantitative recombinant protein standards (qRePSs). The DBSs were subjected to three simulated and realistic shipping environmental conditions and stored in two different types of containers to assess sample stability. The results suggest that shipping conditions and storage at high temperatures often observed during summer have an impact on protein identification and quantification results, highlighting the need for further research to ensure biomarker reliability for dispersed microsampling.

## Graphical abstract

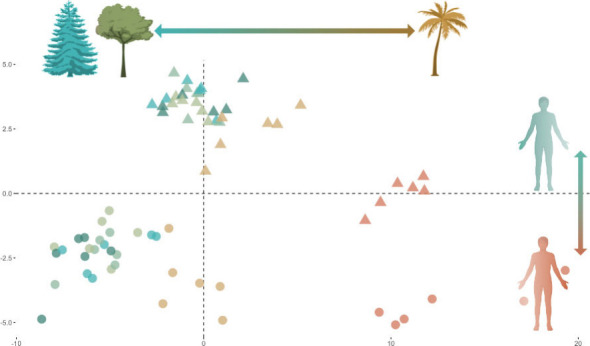

## Introduction

Blood is a proximal fluid and is in contact with all our organs, acting as a reservoir for biomolecules. Thus, through the use of molecular techniques, it can be leveraged to provide information about a person’s physiological status. Furthermore, this biofluid is also readily available. These characteristics make it a primary target for therapeutic interventions.^1^

In modern medicine, venipuncture has been one of the most common invasive procedures to draw blood from individuals.^2^ While this technique is usually innocuous, complications such as peripheral nerve injury or arterial puncture can ensue.^3,4^ Additionally, it must be performed by healthcare professionals using specialized equipment on site. These characteristics make venipuncture suboptimal for population-wide screenings, as well as long-term monitoring or longitudinal studies that require frequent sampling.

Microsampling offers a compelling solution to these drawbacks. Currently, microsampling devices enable the use of small volumes of capillary blood which can be obtained by minimally invasive finger-pricking. This can be done both offsite and without the need for a medical professional. The samples can then be easily transported and stored in the form of dried blood spots (DBSs). While the use of DBSs for large screening initiatives started well over half a century ago,^5^ new volumetric microfluidic devices have now also enabled DBS microsampling of precise volumes.^6,7^ For instance, there are currently several options in the market for microsampling devices that consistently collect 10 μL of capillary blood.

Despite their small volumes, these samples contain important biological information, which state-of-the-art omics technologies increasingly enable us to extract. One of such technologies is mass spectrometry, which enables the robust analysis of small volumes of dried blood, potentially facilitating remote disease monitoring.^8^ In this context, tandem mass spectrometry is an especially compelling method to quantify protein biomarkers because it offers multiplexing potential, while maintaining high specificity.^9^ Furthermore, the use of quantitative recombinant protein standards (qRePSs), in combination with targeted proteomics, has been shown to offer a highly precise and reproducible option for protein identification and quantification using minute volumes of plasma.^10,11^

The combination of using targeted proteomics and microsampling devices makes it possible to profile an individual’s proteome using DBSs collected at home. This can have important implications, such as more cost-effective screening strategies that lead to earlier detection of disease in larger sectors of the population or simply facilitating especially cumbersome and stressful blood drawing instances, such as time-sensitive (e.g., time of the day, onset of specific symptoms) or long-term repeated sampling.^7^

While proteomics and volumetric microsampling can offer a synergistic advantage, the reliability of the molecular data obtained with this mode of sampling still has to be investigated and validated.^7^ Here, we investigate the stability of targeted peptides from samples collected on dried blood microsampling device CapitainerB by simulating practicalities of the shipping process from their at-home collection to their molecular analysis. The shipping environment is hard to control, and the protein stability may be compromised. These microsampling devices were developed using whole blood samples. Blood-derived biofluids are the most commonly used samples for biomarker quantification in mass spectrometry-based proteomics, with plasma being the predominant matrix. The device is based on Capitainer’s qDBS technology, which uses polymer microfluidics and water-soluble membranes to get an exact volume of blood. The user deposits a drop of blood, typically from a finger prick, onto the inlet. The blood fills up the metering channel, traveling to a paper disc, where it dries and forms a DBS. The aim of this study was to explore, using targeted peptide measurements as proxies for protein abundance, how DBS samples are affected after the initial at-home sample collection and how such changes may influence LC-MS/MS-based readouts.

## Methods

### Collection of Dried Blood Samples

Samples were collected from two deidentified donors (*n* = 36, each) following CapitainerB’s instructions for use (2022). Each individual took these samples in a single session using multiple finger pricks. The discs with the samples were left inside their microsampling device CapitainerB to dry at room temperature and then stored at −20 °C. No additional packaging or protection was used during this step.

Sample collection followed the ethical agreement DNR 2021-04164, Development of diagnostic methods based on microsampling of blood, plasma, and interstitial fluid.

### Temperature Treatments

The dried blood microsampling device CapitainerB containing DBS samples from each individual was randomly distributed and subjected to three different shipping simulations (*n* = 12 DBS per individual per treatment, resulting in *n* = 24 DBSs per treatment) modeled after the ISTA 7D 2007 test sequence,^12^ using the environmental test chamber ESPEC SH-642 (ESPEC North America, Inc., Denver, CO, USA) (Table S1): (A) Tropical scenario with a temperature range of 22 to 60 °C, (B) Polar scenario with a temperature range of −30 to 18 °C, and (C) Continental scenario with a temperature range of −10 to 18 °C. All of them had a sustained humidity of 70%. Within each treatment group, the same number of samples per individual were put into Capitainerʼs drying pouch or into resealable sliding channel plastic storage bags (ziploc bags) together with a desiccant bag before the simulation (*n* = 6 DBSs per treatment-storage group per individual, resulting in *n* = 12 DBSs per treatment-storage group). The idea behind all treatments was to simulate sufficiently different scenarios that a DBS sample could be realistically expected to go through from the moment of collection with a microsampling device to the moment of its analysis. A schematic representation of the sample distribution is in Figure S1.

### Sample Preparation

The blood-spotted discs were extracted from the cards using tweezers, and DBSs were eluted by suspending each disc in 400 μL of 0.01% sodium deoxycholate (SDC) and 1× PBS in an Eppendorf tube and incubating at room temperature for 2 h, keeping them in constant movement using an ELMI Intelli-MixerRM-2L (MSE Supplies LLC, Tucson, AZ, USA). Total protein concentrations in the extract were measured using Bio-Rad Protein Assay Kit II #5000002 (Bio-Rad, Berkeley, CA, USA) and the Multiskan GO spectrometer (Thermo Fisher Scientific, Santa Clara, CA, USA).

A modified version of the Human Disease Atlas’s protocol for plasma digestion with vacuum-dried qRePS, adopted from Geyer et al.,^13^ was used. Targeted peptides were quantified using a mix of qRePSs aliquoted at physiological-like levels into 96-well plates, vacuum-dried at 35 °C for 16 h, and stored at −20 °C.^10^ This prealiquoted and dried panel of qRePSs was resuspended by addition of 1× PBS. The resuspended qRePSs were used to spike sample eluates containing 60 μg of total protein content, such that equal amounts of qRePS peptides were added to all samples prior to reduction, alkylation, and digestion. SDC (Sigma-Aldrich, St. Louis, MI, USA) and dithiothreitol (DTT) (Sigma-Aldrich, St. Louis, MI, USA) were added for a final concentration of 0.66% (w/v) SDC and 10 mM DTT. This mixture was incubated at 37 °C for 60 min, during which time samples underwent a reduction process. This was followed by a 30 min alkylation in the dark using chloroacetamide (CAA) (Sigma-Aldrich, St. Louis, MI, USA) at a final concentration of 50 mM. Protein digestion was performed overnight using Trypsin (Thermo Fisher Scientific, Santa Clara, CA, USA) at a 1:25 enzyme-to-substrate ratio. Digestion was quenched with trifluoroacetic acid (TFA, Sigma-Aldrich, St. Louis, MI, USA) to 0.5% (v/v).

Half of the total volume of each sample, corresponding to approximately 30 μg of protein, was extracted using a solid-phase extraction with C18 StageTips packed in-house.^14^ This required activating the C18 matrix (Supelco, Sigma-Aldrich, St. Louis, MI, USA) with 100% acetonitrile (ACN), equilibrating with 0.1% formic acid (FA), loading the samples, washing with 0.1% FA, and eluting the digested peptides with an 80% ACN, 0.1% FA solution. Eluates were vacuum-dried at 45 °C for 70 min and stored at −20 °C until analysis.

### Sample Analysis Using SRM

The assay development and quantitative analysis were performed using an UltiMate 3000 nano-LC system (Thermo Fisher Scientific, Santa Clara, CA, USA) equipped with an EASY-Spray ion source and coupled to a TSQ Altis mass spectrometer (Thermo Fisher Scientific, Santa Clara, CA, USA). The samples were loaded onto an Acclaim PepMap 100 trap column (75 μm × 2 cm, C18, 3 μm, 100 Å, Thermo Fisher Scientific, Santa Clara, CA, USA) and washed with 1% Solvent B (95% ACN, 0.1% FA, water) at a flow rate of 15 μL/min for 0.75 min. Peptide separation was performed with an analytical PepMap RSLC C18 column (150 μm × 15 cm, 2 μm, 100 Å, Thermo Fisher Scientific, Santa Clara, CA, USA) and a gradient of Solvent B from 1% to 30% at 3 μL/min for 29.25 min. The columns were washed three times with 95% Solvent B for 30 s, followed by a single wash with 1% Solvent B, and re-equilibrated with 1% Solvent B for 1.4 min. Sample loading, analysis, and column re-equilibration made up a total cycle time of 35 min for plasma quantification and 15 min for method development. Temperatures were set at 40 °C for the analytical column, 60 °C for the column oven, and 10 °C for the autosampler.

The SRM method followed a scheduled inclusion list with precursors and transitions targeting the 108 proteins and 205 peptides listed in Table S2. Precursor ion mass-to-charge ratios, targeted transitions, average measured retention times, and collision energies are detailed in Table S3. A uniform retention time window of 2 min was applied to all precursors. Complete SRM methods, chromatograms, and raw data are provided via Panorama. The assay library was provided by the Human Disease Blood Atlas. Ratios to standard were calculated as the signal intensity of the endogenous (light) peptides divided by the corresponding qRePS (heavy) peptide signal, which served as a one-point internal calibrant.

### Data Preprocessing

Skyline (beta version 23.1.1.459)^15^ was used for peak integration and the initial quality control of the raw mass spectrometry data. Reports annotated with replicates, peptides being measured, proteins they represent, and ratios-to-standards were exported from Skyline as CSV files. Bioinformatic analyses were performed in R (version 4.5.2).^16^ Quality control of the data was based on the qRePS signals where data points with a library dot product (dotp) or a peptide peak found ratio (rdotp) below 0.8 were excluded as well as peptides identified in less than 50% of the samples.

### Differential Expression Analysis

Two-sided *t* tests were applied at the peptide level to estimate differential expression between experimental groups using ratios to standards. For each comparison, *p*-values were adjusted for multiple testing using the Benjamini-Hochberg false discovery rate (FDR) procedure across all peptides tested with significance defined as *q* < 0.05. Fold changes were calculated as the ratio of the mean normalized peptide abundance in the test group relative to the reference group. Volcano plots summarize the fold changes and FDR-adjusted *p*-values (*q*-values) for each comparison. Although statistical testing was performed at the peptide level, volcano plots are annotated using the corresponding protein names for clarity and interpretability.

To assess the consistency of peptide-level quantitative measurements across shipping conditions, pairwise correlation analyses were performed using the median peptide ratios-to-standards. Prior to analysis, extreme values were removed using an interquartile range-based filter (1.5 × IQR). For each peptide, median ratios-to-standards were calculated per patient and treatment. Pairwise comparisons between treatments were evaluated using Pearson correlation coefficients calculated on a per-patient basis. Two-sided significance testing for correlation was performed using Pearson’s correlation test with missing values excluded pairwise.

The nonparametric Kruskal–Wallis test was used to compare peptide abundance between treatment-storage groups. Post hoc pairwise comparisons between treatment-storage groups were performed using two-sided Wilcoxon rank-sum tests (also known as Mann–Whitney U test). *p*-values from all pairwise comparisons were adjusted for multiple testing using the Benjamin-Hochberg procedure with significance defined as *q* < 0.05.

### Principal Components Analysis

Missing peptide ratio-to-standard values were imputed using a hot-deck approach from the VIM package (v 6.2.2)^17^ with imputation performed across all variables. PCA was then applied to the imputed data set using the prcomp from R stats (v 4.5.2),^16^ with samples treated as observations and peptides, as variables. The data were centered and scaled prior to decomposition.

### Data Visualization

Graphs were generated with R (v 4.5.2),^16^ using the ggplot2 (v 3.5.0),^18^ ggpubr (v 0.6.0),^19^ ggstatsplot (v 0.12.2),^20^ ggbeeswarm (v 0.7.2),^21^ ggrepel (version 0.9.5),^22^ and factoextra (v 1.0.7)^23^ packages. Icons from Figures 1, S1, and S2 were created using BioRender.com, Affinity Designer 2, and generative OpenAI.

**Figure 1:**
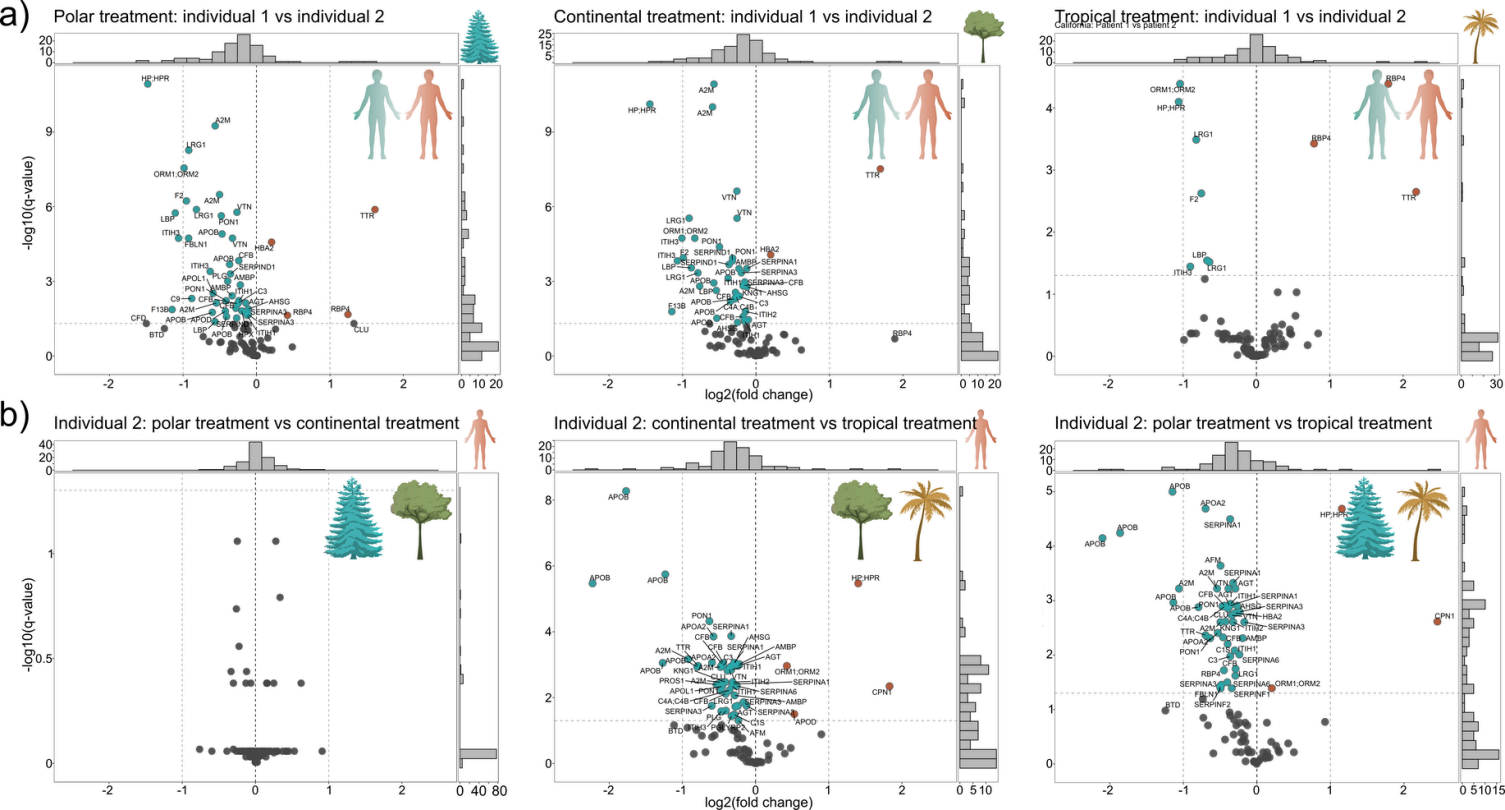
Differential expression analysis using two-sided *t* test at peptide level and the Benjamini-Hochberg procedure for multiple hypothesis correction. The volcano plots summarize the peptides that show significantly different levels a) between the individuals within the different treatments; b) between the treatments for individual 2. The peptides below the established level of significance (*q*-value < 0.05) are marked with red when the case (individual 2 or warmer treatment) shows higher levels than the reference (individual 1 or colder treatment) and blue when the case shows lower levels than the reference. Peptides are represented by the gene symbol of the protein to which they belong for interpretability.

## Results

For this study, 205 peptides from 108 target plasma proteins were analyzed in 72 blood samples from two individuals collected with dried blood microsampling devices CapitainerB. Peptides from the targeted proteins were quantified using internal protein standards in combination with targeted mass spectrometry. The result files obtained from the SRM read out were subjected to quality control after which 96 peptides from 58 proteins were used for downstream bioinformatic analyses. Multiple peptides per protein were quantified. While this can provide redundancy in protein coverage, formal assessment of peptide-to-peptide consistency within proteins was not performed in this study.

### Identification of Protein Level Patterns through Differential Expression Analysis

Differential expression analyses (DEAs) were used to compare peptide-based proteomic profiles. All differential expression analyses were performed using peptide ratios-to-standards such that comparisons reflect differences in peptide abundance after normalization to internal standards. While statistical testing was conducted at the peptide level, volcano plots are labeled by the proteins to which the differentially expressed peptides belong. The summaries of the proteins whose peptides show significantly different levels are listed in Figure 1. These include (i) an individual-to-individual comparison within each treatment, using individual 1 as the reference and individual 2 as the case (Figure 1a), and (ii) an intraindividual comparison using the lower-temperature treatment as the reference and the higher-temperature one as the case (Figures 1b and S2). This analysis showcased a pattern of protein instability for the Tropical treatment. This resulted in the differences between individuals being reduced at higher temperatures (Figure 1a) as well as in considerably different peptide profiles for a single individual when comparing replicates subjected to the Tropical treatment to replicates subjected to either of the other two (Figure 1b). This second pattern reveals a loss of signal intensity for the Tropical treatment in most of the significantly different peptides (Figure 2). The two-sided *t* test results (fold changes and *q*-values) for each comparison are detailed in the Supporting Information (Table S4). These results were replicated on a separate independent experiment that made use of the same technology with two different temperature treatments (Figure S3).

**Figure 2:**
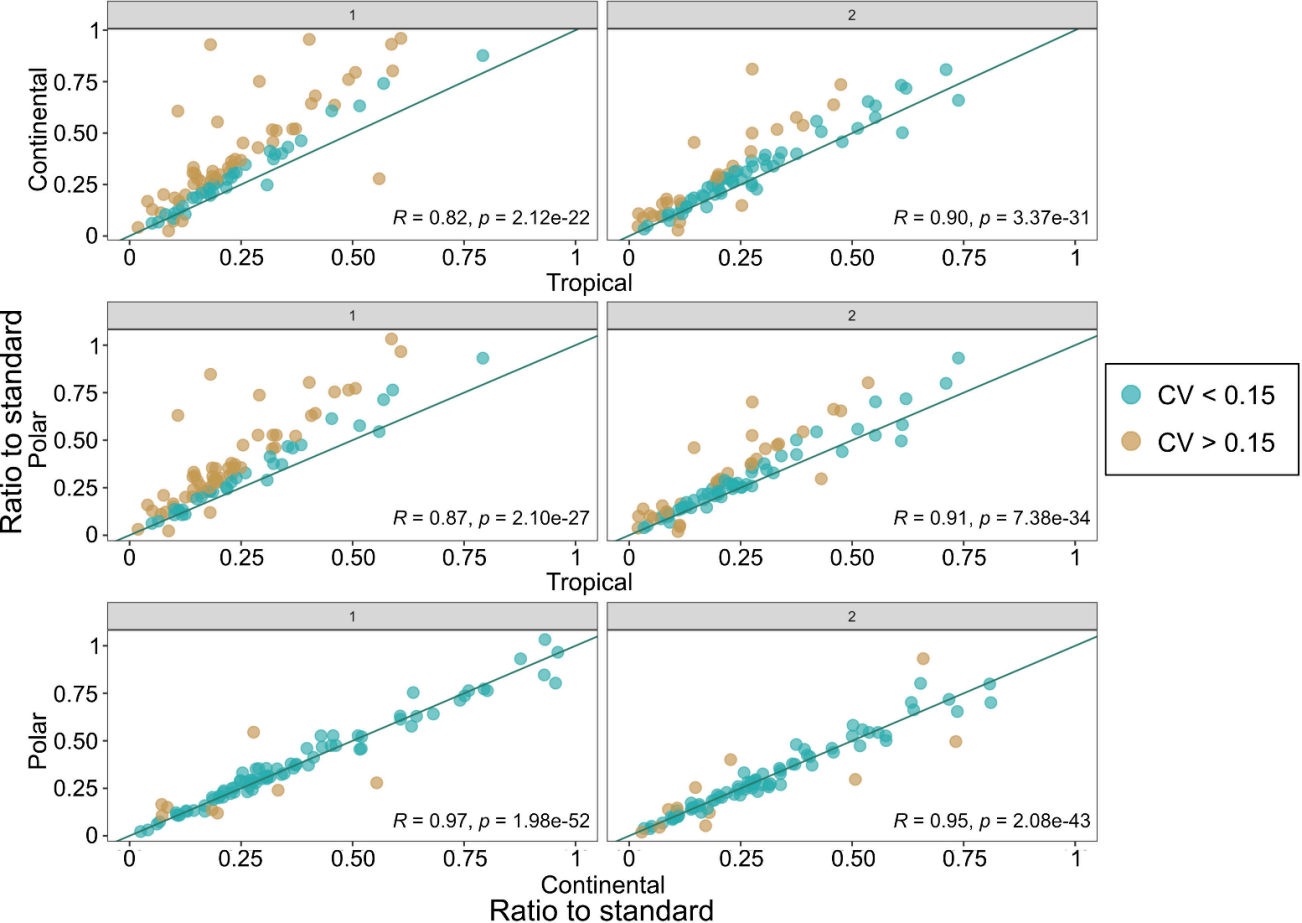
Correlation of peptide levels between treatments. Scatter plots of the median ratio-to-standard values for individual peptides across the Tropical, Continental, and Polar treatments for individual 1 (left) and individual 2 (right). Each panel compares two treatments with their level of correlation indicated by the Pearson Correlation Coefficient (*R*) and its significance indicated by the *p*-value (*p*), highlighting intraindividual variability. Peptides with a coefficient of variation greater than 15% of what would be the perfect correlation (*y* = *x*, solid blue line) are marked as outliers (brown), while values with more than 1.5× IQR were excluded.

Furthermore, while all treatments used the same level of humidity (70%), different storage types were used to protect the samples. One of them, provided by the company, is a cardboard drying pouch, which mainly protects the discs from dust or contact with other objects. The other type consisted of a ziploc bag with a pack of desiccant, expected to exert increased protection from humidity. The results indicate that the effects of temperature on the samples are less dramatic with increased protection against humidity (i.e., using a ziploc bag and a desiccant) (Figure 3). Nevertheless, this increased protection did not seem to be enough to completely prevent the impact of the environmental conditions on peptide levels. From the 96 analyzed peptides, 71 had a significant *q*-value (<0.05) in the nonparametric Kruskal–Wallis test comparing treatment-storage groups (Table S5 details these and the Wilcoxon rank-sum posthoc tests *q*-values by peptide). The results from two independent experiments, one in which this protection was doubled (the dried blood microsampling device CapitainerB was stored in a ziploc bag with a desiccant pack within a second ziploc bag and desiccant pack) (Figure S3) and one where the DBS discs were put into a 96-well plate and heated inside a PCR machine using a temperature gradient program where the humidity was not adjusted (Figure S4), showed similar patterns, pointing to humidity only as an intensifier of the impact of heat.

**Figure 3:**
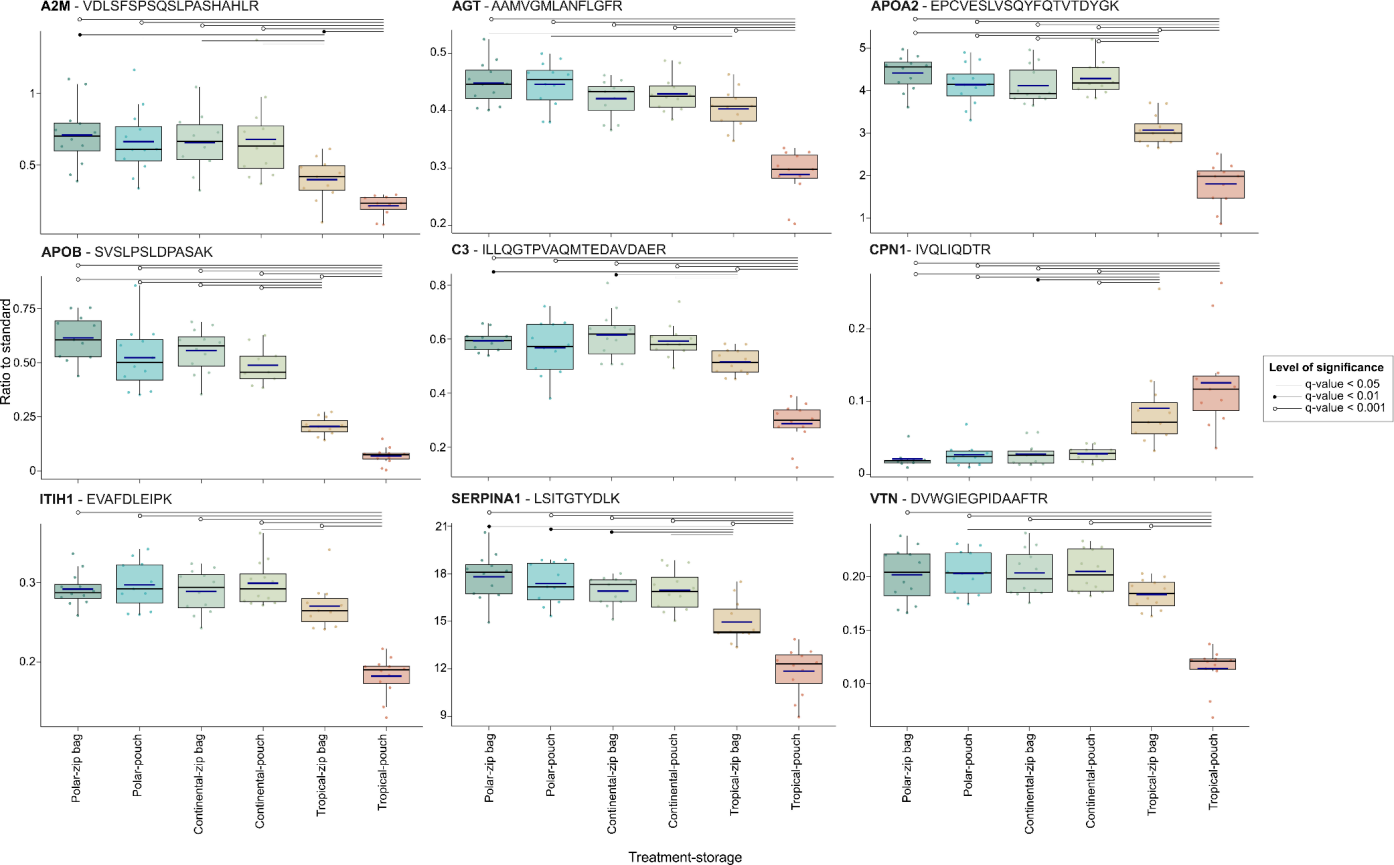
Boxplots of example peptides with significantly different levels between treatments and storage types. Each dot represents the ratio-to-standard per peptide and replicate. From these peptide values, the median, first, and third quartiles are summarized by the boxplots, and the whiskers mark the minimum and maximum values per group, while the thicker dark blue horizontal line indicates the mean. Samples are grouped by the interaction between treatment and storage type (Polar-ziploc bag, Polar-pouch, Continental-ziploc bag, Continental-pouch, Tropical-ziploc bag, Tropical-pouch). Each treatment-storage group had a total of *n* = 12 DBS samples with *n* = 6 DBSs per individual (unless protein measurements did not pass the quality control for a specific replicate). On top of each plot, the horizontal lines indicate which pairwise comparisons are significant. *p*-values were calculated using the posthoc Wilcoxon rank-sum test and corrected with the Benjamin-Hochberg procedure. The line style indicates the level of significance, where plain is significant (*q*-value < 0.05), left black circle is very significant (*q*-value < 0.01), and left white circle is highly significant (*q*-value < 0.001).

### Principal Component Analysis of Sample Similarity

PCA was performed to visualize global patterns in peptide-based proteomic profiles across individuals, treatments, and storage (Figure 4). It was conducted on imputed ratios-to-standards, such that each peptide’s ratio-to-standard contributes to the multivariate representation of the samples. The resulting PCA plot shows a clear separation between the two individuals for all treatment-storage subgroups, primarily along the *y*-axis (Dim-2), reflecting individual-specific differences in peptide profiles. Along the *x*-axis (Dim-1), samples subjected to the lower-temperature Polar and Continental treatments cluster together, and samples exposed to the higher-temperature Tropical treatment separate from the rest. This separation is already apparent for the Tropical treatment samples stored in the more humidity-resistant ziploc bag with desiccant and becomes markedly more pronounced for those stored in the less protective cardboard pouch. These patterns support the findings from the differential expression analyses, indicating that elevated temperature adversely affects the reliability of peptide-based proteomic profiling and that the impact is exacerbated when protection against humidity is reduced.

**Figure 4:**
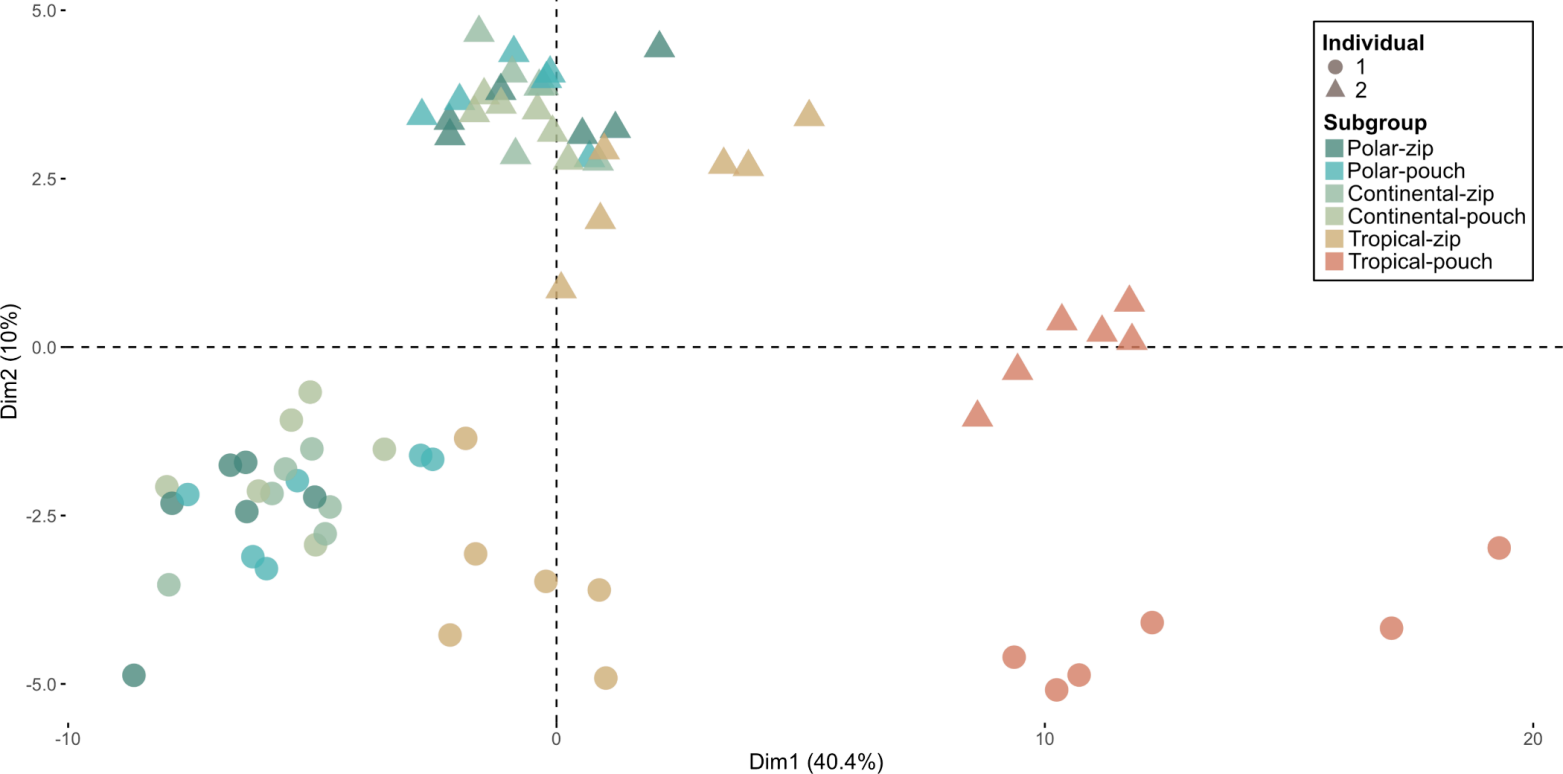
Principal component analysis (PCA) of peptide-level proteomic data. The graph shows the first two principal components (PC1 and PC2). Each point represents one DBS sample. Points are colored according to the storage-treatment interaction, while shapes indicate the individual from which the sample was derived.

The ratio-to-standard coefficients of variation (CVs) were obtained per peptide for each of the 12 individual, treatment, and storage subgroups (Table S6). These coefficients were obtained from biological replicates (multiple samples from the same individuals) instead of technical replicates. However, they still reflect a decrease in robustness associated with high-temperature conditions and lessened protection against humidity with the smallest median CV (13.80) belonging to one of the subgroups where samples were subjected to the continental treatment stored in ziploc bags and the highest median CV (28.87) belonging to one where samples were subjected to the Tropical treatment in cardboard pouches (Table S7).

## Discussion

The targeted assay panel used in this study was originally developed and optimized for plasma samples. Nevertheless, 96 peptides from 58 proteins met the quality control criteria when transferred to DBS samples, enabling downstream analyses. Initially, the expectation for this study was to observe variability attributable to technical variability, such as that seen between the Continental and Polar conditions. However, the findings point to a lack of robustness in the peptide-based proteomic profiles for the increased temperature treatment. This was observed despite having sampled all replicates in a single session per individual and having prepared them for the mass spectrometry-based analysis simultaneously after the treatments. While this Tropical treatment simulates an extreme scenario that might seem improbable, studies dating back to the nineties, such as Black and Layloff’s (1996)^24^ and Okeke et al.’s (1997)^25^ or more recent ones such as Laven-Law et al.’s (2023)^26^ have shown that pharmaceutical products and samples can be subjected to temperatures over 50 °C and even over 60 °C during the shipping process when left in metal sheet mailboxes and vehicles standing in the sun. This is a very plausible scenario when individuals participating in decentralized studies drop off their sample for analysis in the nearest mailbox. Thus, while our simulation protocols were modeled after the ISTA 7D 2007 shipping standard,^12^ which only goes up to 40 °C, we chose to elevate the temperature to address these previous findings. On the other hand, while the drying pouch is meant as an additional cover to ensure that the dried blood microsampling device CapitainerB is allowed to be ventilated when put into shipping envelopes rather than as a stand-alone shipping container itself, this design better allowed for a comparison between reduced and increased protection.

While it is well understood that proteins lose stability with elevated temperatures, these results raise critical considerations. Microsampling devices leverage DBS technology, which is designed to minimize unwanted protein interactions by stabilizing proteins in their dried state. This feature has the power to facilitate longitudinal studies and expand healthcare access to remote communities. Previous studies have already delved into the molecular analysis of DBSs using mass spectrometry to evaluate sample stability.^27–29^ Chambers et al.^27^ concluded that the concentrations for most of the proteins tested were stable for at least 10 days when stored in a range of temperatures from −20 to 37 °C. Shifting the focus to the need for cold chain logistics, Eshghi et al.^29^ tested robust protein quantification across extended periods (up to 57 days) for temperatures spanning from −20 to 40 °C. They found a decrease in quantitative precision for DBS samples exposed to higher temperatures for multiple weeks.

However, these studies focus mainly on the effect of storage by prioritizing the study of sample stability over time in conditions that might be expected in a laboratory environment. Instead, the present study focuses completely on sample shipping, an intrinsically variable and less controlled process that can expose DBS samples to short-term but more extreme temperature fluctuations. Thus, this study includes ranges from −30 to 60 °C, which exceed the upper limits examined in previous work but have been reported in real-world shipping scenarios. While such conditions may represent an extreme case, they are relevant for at-home sampling workflows and highlight the importance of considering transient, high-temperature exposures that are not typically addressed in storage-focused studies. This is especially relevant when considering the aim of microsampling devices to be used to improve healthcare access in remote regions worldwide. Thus, while DBS can be a robust source of molecular information after extended storage in milder, more stable conditions, the results from our differential expression analyses point to a destabilizing effect of high temperatures when using microsampling devices, even after taking robust measures against humidity. Furthermore, it is unfeasible to regulate the environmental conditions the devices are subjected to from the moment of sample collection to the point of laboratory analysis. If the assumption of DBS sample stability does not hold, it undermines the reliability of microsampling devices for real-world applications, requiring further investigation.

Consistent with the differential expression analyses, the PCA of the peptide ratios to standards further highlighted the impact of treatment and storage conditions on overall sample similarity. While samples from the two individuals remained separated along the second principal component (*y* axis), those samples subjected to higher temperatures did not cluster with the others. Once again, the fact that the separation was more pronounced for samples stored in the less protective drying pouch compared to those in the ziploc bag with desiccant supports the conclusion that heat, humidity, and their interaction compromise the reliability of peptide-based proteomic profiles.

Studies such as those by Fredolini et al.,^6^ who profiled proteins related to SARS-CoV-2 infection from home-sampled DBSs, exemplify the expanding role of microsampling in decentralized and population-scale studies. These investigations rely on participants collecting and shipping their samples from uncontrolled, real-life environments. While these protocols reflect practical advances in public health research, our findings suggest that the stability of the proteome under variable shipping conditions remains a vulnerable point in the chain of the sample integrity. If shipping environments compromise protein stability, especially under elevated temperatures, then the data quality of at-home sampling-based studies could be impacted in ways not currently accounted for. This highlights a gap in the validation of microsampling workflows under fieldlike conditions. As large-scale proteomics technologies are applied to even larger cohorts, it is of the utmost importance to adapt the shipping environment to ensure that cards are stored in a controlled environment. This would potentially limit the sampling flexibility by ensuring that the resulting data do not suffer from seasonal or geographic effects when samples are exposed to biases that relative quantification cannot compensate for nor identify.

Notably, in targeted mass spectrometry approaches like the one employed here, we rely on fragmented peptides rather than intact proteins. The destructive sample preparation process, which includes denaturation, means that protein integrity is not a requirement for detection, as opposed to affinity-based assays that might depend on native protein structures. In theory, prior denaturation due to high temperatures should either have minimal impact on results or reveal patterns resembling upregulation. Instead, we have observed a tendency to reduced peptide signals, which suggests that some underlying, not yet understood mechanism, such as undesired interactions between the sample and device, can alter the individual’s proteome profile. This finding indicates the need for further studies to assess the robustness of microsampling devices. While samples can be reliably shipped under controlled conditions, enabling broader use of these devices will require deeper understanding of their performance in warmer or less regulated environments. Addressing these challenges is essential to realize the full potential of decentralized sampling and ensure equitable access to advanced diagnostics across diverse global settings.

## Supplementary Material





## Data Availability

Raw data and Skyline documents of method development and quantification are available on Panorama Public^30^ (https://panoramaweb.org/env-cap.url).

## Notes

Supplementary Figure 1, a schematic representation of the experimental design and sample allocation. Supplementary Figure S2, a volcano plot that summarizes the proteins that show significantly different levels between the treatments for individual 1. Supplementary Figure S3, the results of an additional, supporting experiment with a volcano plot that summarizes the differential expression analysis between two temperature treatments. Supplementary Figure S4, the results of a second additional experiment with boxplots of proteins with significantly different expression levels between these treatments (PDF)
Supplementary Table S1, additional experimental details about the simulation conditions in the test chamber ESPEC SH-642. Supplementary Table S2, the peptides and proteins targeted by our SRM method. Supplementary Table S3, additional parameters for the SRM method, including precursor ion *m*/*z*, targeted transitions, measured average retention times, and collision energies. Supplementary Table S4, the fold-changes and *q*-values of the *t* tests for the intrapatient and interpatient comparisons. Supplementary Table S5, the *q*-values of the Kruskal–Wallis and Wilcoxon rank-sum posthoc tests by protein. Supplementary Table S6, the coefficients of variation of the ratios-to-standards per peptide and individual, treatment, and storage subgroup. Supplementary Table S7, the median coefficient of variation of the peptide ratios-to-standards per individual, treatment, and storage subgroup (XLSX)

## ○

ImmunoIVD AB, Nacka, Sweden

## Abbreviations

DBS: dried blood spot
qRePS: quantitative recombinant protein standards
SRM: selected reaction monitoring
LC-MS/MS: liquid chromatography tandem mass spectrometry
PBS: phosphate buffered saline
SDC: sodium deoxycholate
DTT: dithiothreitol
CAA: chloroacetamide
TFA: trifluoroacetic acid
ACN: acetonitrile
FA: formic acid
FDR: false discovery rate
DEA: differential expression analysis
FC: fold change
