# Supplementary material for: Shipping Conditions May Impact the Reliability of At-Home Microsampling Devices
Source: J Proteome Res. 2026 Jul 14;25(8):3817–25. doi: 10.1021/acs.jproteome.6c00117 (PMC13459527; doi:10.1021/acs.jproteome.6c00117)
Supplement: Supplementary file 1 [file pr6c00117_si_001.pdf]

Supplementary Information

Shipping conditions may impact the reliability of at-home microsampling devices

Andrea Villanueva Raisman[1], Jakob Naess[1 †], David Kotol[2], Fredrik Edfors[1]*

[1] SciLifeLab, Department of Protein Science, Division of Systems Biology, School of Chemistry, Biotechnology and Health, KTH Royal Institute of Technology, 171 65 Solna, Sweden

[2] ProteomEdge AB, 106 91 Stockholm, Sweden

*corresponding author, fredrik.edfors@scilifelab.se

Supplementary Figures
Figure S1. Schematic overview of study workflow.
Figure S2. Peptide-level volcano plots for Individual 1 (two-sided t-test, Benjamini–Hochberg FDR).
Figure S3. Peptide-level volcano plots across treatments (combined and per individual; two-sided t-test, Benjamini–Hochberg FDR).
Figure S4. Peptide ratio-to-standard boxplots by temperature for proteins showing significant treatment effects.

Supplementary Tables (XLSX)
Table S1. Settings for shipping simulations
Table S2. List of protein and peptide targets
Table S3. List of targeted peptides with their mass-to-charge ratio, transitions, retention times and collision energies
Table S4. Two-sided t-tests results per peptide for inter- and intra-patient comparisons using ratios-to-standards
Table S5. Q-values by peptide from the Kruskal-Wallis and Wilcoxon rank-sum post-hoc tests corrected for multiple testing using the Benjamin-Hochberg procedure
Table S6. Coefficients of variation by peptide for each of the twelve individual, treatment and storage subgroups
Table S7. Median coefficient of variation for each of the twelve individual, treatment and storage subgroups

Supplementary figures

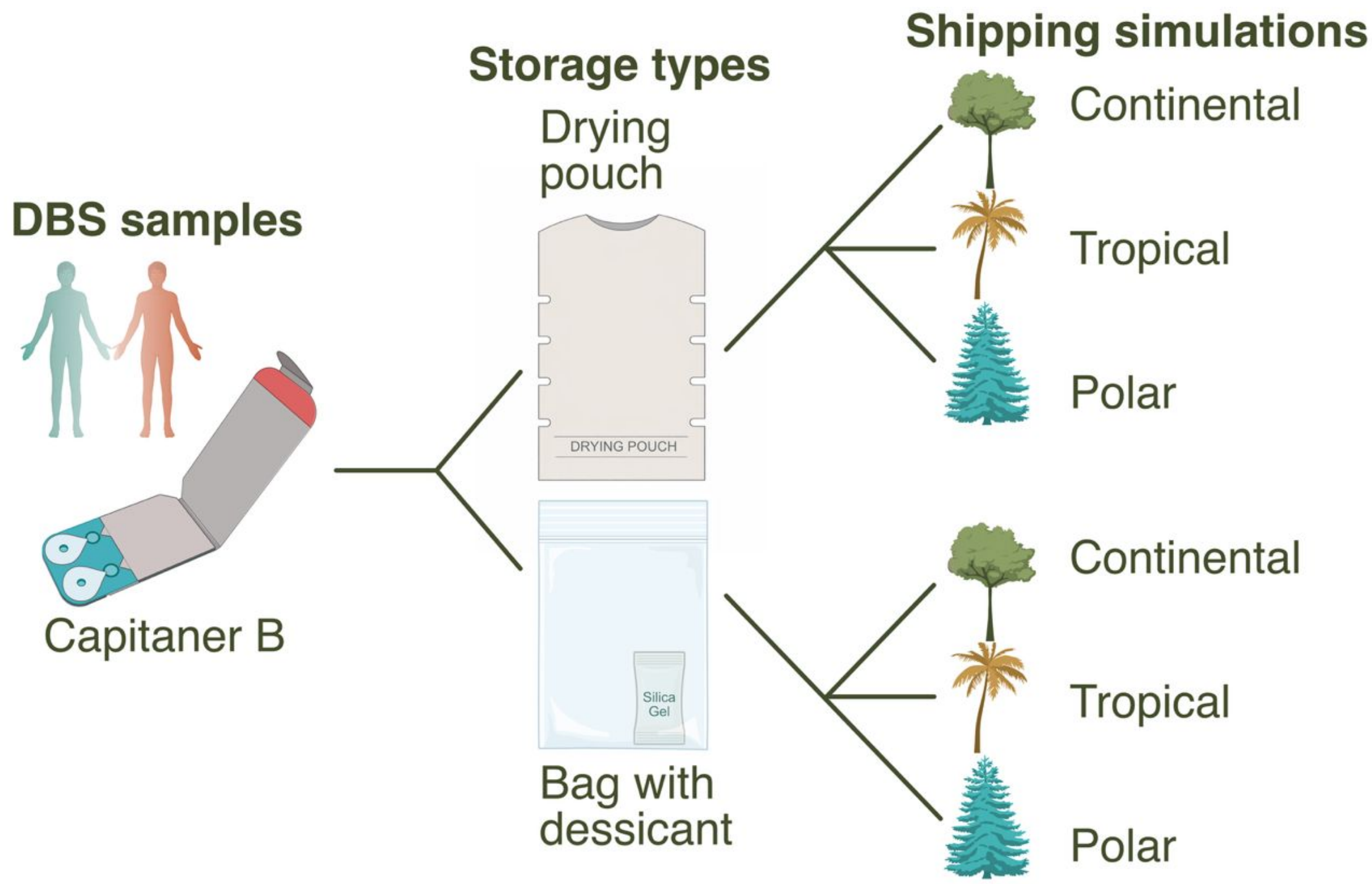

Figure S1. Experimental design and sample distribution. Schematic overview of the study workflow showing sample allocation. Two donors (patients) provided an equal number of dried blood spots (DBS) (n=36 per individual, n=72 in total). Samples from each donor were equally split into two storage types, and each storage type was further divided across three treatment

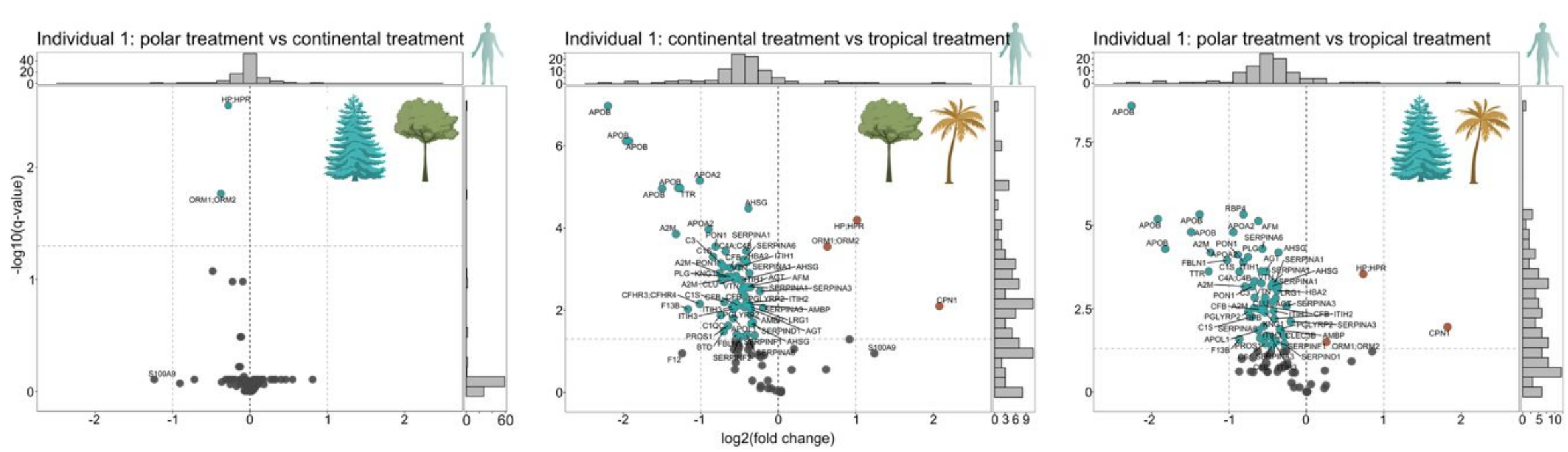

conditions (Tropical, Continental, Polar).

Figure S2. Differential expression analysis using two-sided t-test at peptide level and the Benjamini-Hochberg procedure for multiple hypothesis correction. The volcano plots summarize the peptides that show significantly different levels between the treatments for the individual 1. The peptides below the established level of significance (q-value < 0.05) are marked with red when the case (warmer treatment) shows higher levels than the reference (colder treatment); and blue when the case shows lower levels than the reference. Peptides are represented by the gene

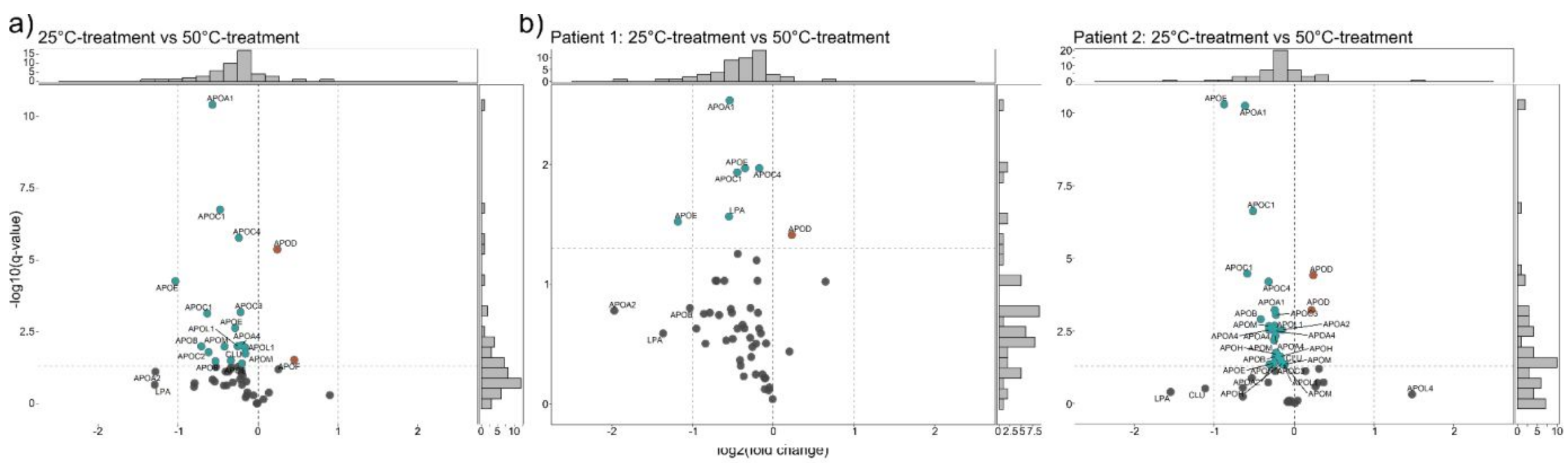

symbol of the protein to which they belong. Q-values and fold-changes per peptide detailed in **Table S4**.

Figure S3. Differential expression analysis using two-sided t-test at peptide level and the Benjamini-Hochberg procedure for multiple hypothesis correction. Volcano plots summarizing the proteins that show significantly different levels between a) treatments for both patients simultaneously and between b) treatments for patient 1 (center) and patient 2 (right) with the fold change (FC) on the x-axis and the significance (q-value) on the y-axis. Two-sided t-tests were applied for each peptide and q-values obtained after Benjamin-Hochberg FDR-corrections. The peptides below the established level of significance (q-value < 0.05) are marked with red when

the case (50˚C-treatment) shows higher peptide levels than the reference (25˚C-treatment) and blue when the case shows lower levels than the reference. Peptides are represented by the gene

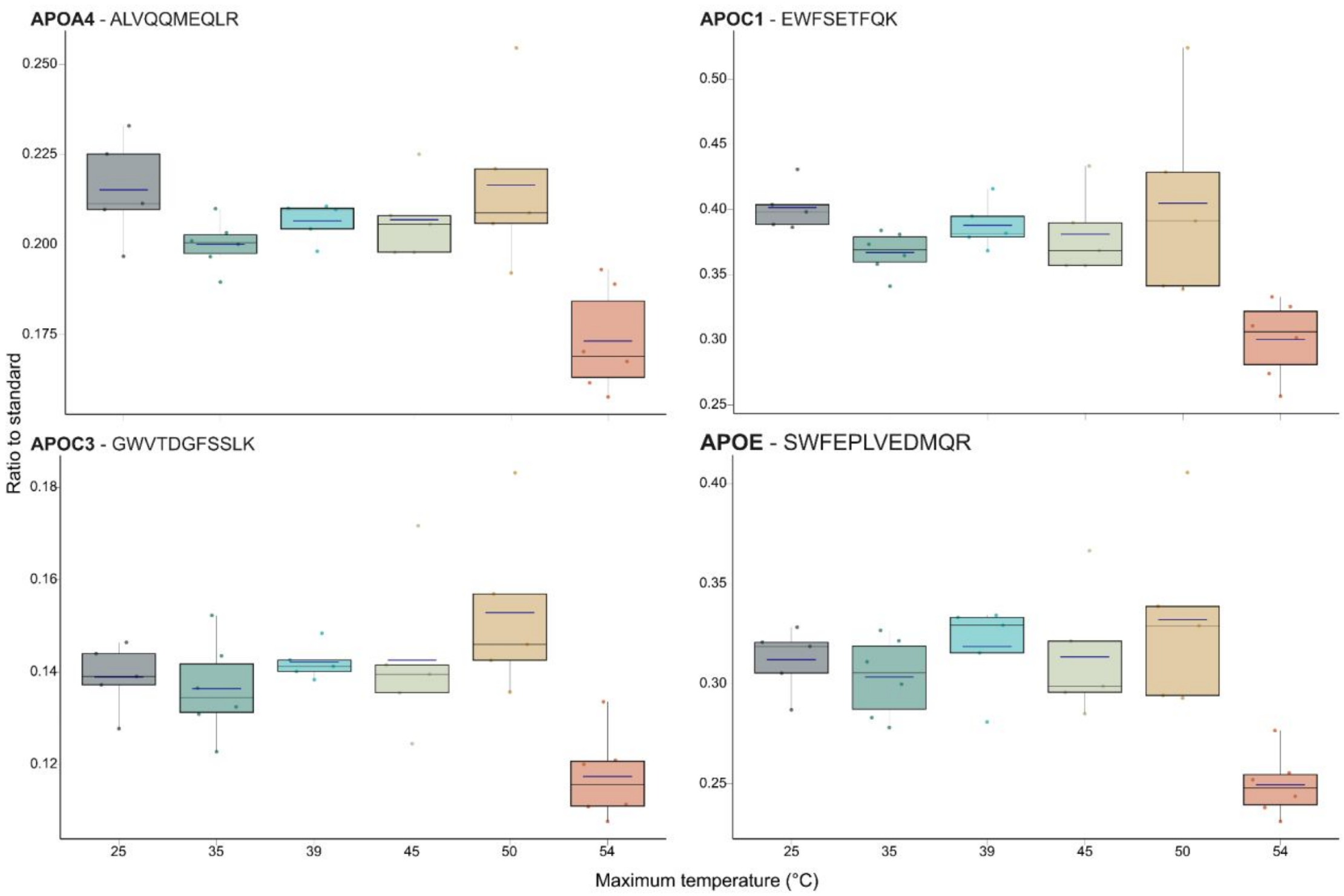

symbol of the protein to which they belong.

Figure S4. Boxplots of the proteins with significantly different levels between treatments. Each dot represents a sample, with samples grouped by the temperature treatment they were subjected to (maximum temperature on the x-axis; 25˚C, 35˚C, 39˚C, 45˚C, 50˚C, 54˚C). The measurement is the ratio to standard (y-axis) of a peptide, whose protein is also indicated on the top. There are up to n = 6 peptide measurements per group, with some groups having fewer for certain peptides due to that measurement not passing the quality controls. The group median, first and third quartiles are summarized by the boxplots, and the whiskers mark the minimum and maximum values per group, while the blue horizontal line indicates the mean. Peptide abundance

between temperature groups was compared using the non-parametric Kruskal-Wallis test. Adjusting for multiple testing using the Benjamin-Hochberg procedure, their q-values were ALVQQMEQLR = 0.021, EWFSETFQK = 0.021, GWVTDGFSSLK = 0.026, SWFEPLVEDMQR = 0.025.
